# Interim report on engineered NK cell trial in lung cancer refractory to immune checkpoint inhibitors

**DOI:** 10.1172/jci.insight.186890

**Published:** 2025-02-04

**Authors:** Miguel A. Villalona-Calero, Lei Tian, Xiaochen Li, Joycelynne M. Palmer, Claudia Aceves, Hans Meisen, Catherine Cortez, Timothy W. Synold, Colt Egelston, Jeffrey VanDeusen, Ivone Bruno, Lei Zhang, Eliezer Romeu-Bonilla, Omer Butt, Stephen J. Forman, Michael A. Caligiuri, Jianhua Yu

**Affiliations:** 1 The Department of Medical Oncology and Experimental Therapeutics, Beckman Research Institute and Comprehensive Cancer Center,; 2 Hematologic Malignancies Research Institute, Department of Hematology and Hematopoietic Stem Cell Transplantation,; 3 Division of Biostatistics, Department of Computational and Quantitative Medicine,; 4 Beckman Research Institute and Comprehensive Cancer Center, and; 5 Beckman Research Institute, City of Hope National Medical Center, Los Angeles, California, USA.; 6 Avita Health System, Galion, Ohio, USA.; 7 CytoImmune Therapeutics, Los Angeles, California, USA.; 8 Institute for Precision Cancer Therapeutics and Immuno-Oncology, Chao Family Comprehensive Cancer Center, Orange, California, USA.; 9 The Clemons Family Center for Transformative Cancer Research, University of California, Irvine, California, USA.

**Keywords:** Clinical trials, Oncology, Innate immunity, Lung cancer, NK cells

## Abstract

BACKGROUND. Non–small cell lung cancer (NSCLC) remains the leading cause of cancer-related mortality, necessitating the exploration of alternate therapeutic approaches. Tumor-reactive or activated-by-cytokine killers (TRACK) are PD-L1^+^, highly cytolytic NK cells derived from umbilical cord blood NK cells and engineered to express soluble IL-15 (sIL15), and these cells show promise in preclinical studies against NSCLC.

METHODS. We assessed safety, persistence, homing, and cytotoxic activity in 6 patients with advanced, refractory, and progressing NSCLC who received a low dose of unmatched, allogeneic, off-the-shelf sIL15_TRACK NK cells. We evaluated NK cell presence and persistence with droplet digital PCR (ddPCR), flow cytometry, and immunofluorescence staining.

RESULTS. sIL15_TRACK NK cells had peak measurements at 1 hour and became undetectable 4 hours after each infusion. Cognate ligands to activating NK cell receptors were found in NSCLC. sIL15_TRACK NK cells were observed in a lung tumor biopsy 7 days after the final infusion, confirming their sustainment and tumor-homing ability. They retained cytolytic function following isolation from the lung tumor. Three of 6 patients achieved disease stabilization on repeat imaging, while the others progressed.

CONCLUSION. Unmatched, allogeneic, cryopreserved, off-the-shelf sIL15_TRACK NK cells express activating receptors, home to tumor sites that express their cognate ligands, and retain cytolytic activity after infusion, underscoring their potential as a therapeutic approach in solid tumors. At low doses, the therapy was safely administered and showed preliminary evidence of activity in 3 of 6 patients with advanced and progressive NSCLC. Additional dose escalation cohorts and coadministration with atezolizumab are planned.

TRIAL REGISTRATION. ClinicalTrials.gov NCT05334329.

FUNDING. Funding was provided by CytoImmune Therapeutics and grants from the National Cancer Institute (CA266457, CA033572, and CA210087).

## Abstract 2

We demonstrate that cryopreserved engineered human NK cells can home to the tumor in lung cancer patients, retain cytotoxic activity, and persist within the tumor seven days after infusion

## Introduction

Despite substantial advances in the prevention, diagnosis, and treatment of lung cancer, it is still responsible for more cancer deaths than any other malignancy. In 2022 alone, lung cancer killed an estimated 1.8 million people worldwide (1). Risk factors for lung cancer extend beyond tobacco use and include environmental and workplace exposures to diesel exhaust, air pollution, genetics, and radon (2). Unfortunately, the cure for lung cancer remains elusive and represents a major unmet medical need across the globe. It is hard to overstate the importance of discovering new therapies for this highly lethal disease.

The most common type of lung cancer is non–small cell lung cancer (NSCLC), which represents 80%–85% of diagnoses (3). Over the last decade, there has been a substantial increase in the number of approved therapies for the treatment of advanced NSCLC stemming from the discovery of immune checkpoints such as programmed cell death-1 (PD-1) and CTLA-4 (4–7) as well as an ever-increasing array of driver mutations with matching targeted therapies (8). The mechanism by which patients with tumors that lack PD-L1 expression can respond favorably to anti–PD-L1 mAb therapy likely involves PD-L1^+^ NK cells in the tumor microenvironment (TME) (9, 10). PD-L1 is induced on the NK cell surface when NK cells recognize tumors. Furthermore, anti–PD-L1 mAb directly activates the PD-L1^+^ NK cell to increase its granule exocytosis and IFN-γ production and further upregulates expression of PD-L1 in a positive feedback loop, thereby amplifying this effect (9). NK cells are capable of trafficking to the lung TME (11, 12). Furthermore, as lung cancer cells become more refractory to standard chemo- and immunotherapy, they often downregulate or lose their MHC class I expression (13), thereby escaping T cell recognition, yet becoming more susceptible to NK cell killing that is enhanced in the absence of MHC class I (14). Finally, NSCLC commonly expresses MICA/B and CD155/CD112 and can also express BAG6/B7-H6 (15–17); these are the ligands for activating receptors NKG2D, DNAM-1, and NKp30, respectively, commonly expressed on human NK cells (18–21). Collectively, these data suggest that activated NK cell therapy may be useful in NSCLC, even in the absence of a chimeric antigen receptor (CAR).

In 2020, a proof-of-concept study was undertaken in which 109 patients with advanced relapsed NSCLC were randomized to be treated with a checkpoint inhibitor alone or a checkpoint inhibitor along with one or multiple infusions of fresh, allogeneic, MHC-unmatched, activated, but otherwise unmodified, NK cells. The latter group demonstrated a significantly prolonged overall survival and progression-free survival, which was most apparent in those patients receiving multiple infusions of the NK cells (22). These data suggest that activated allogeneic NK cells have antitumor activity in patients with NSCLC. However, there was no documentation of the NK cells trafficking to the lung cancer, or what the NK cells might be recognizing in the lung cancer, or evidence of their survival and potency at the site of the lung cancer. Moreover, the absence of any cryopreservation limited the broad application of this therapy.

To overcome the limitations of fresh, unmodified, unmatched, and activated allogeneic human NK cells, it is crucial to explore novel strategies that improve the efficacy, safety, and scalability of NK cell therapies for NSCLC. One potential approach is to engineer allogeneic NK cells to have enhanced functional capabilities followed by ex vivo expansion and successful cryopreservation. An expanded, MHC-unmatched, cryopreserved “off-the-shelf” NK cell product could be delivered on multiple occasions to more than one patient, thereby reducing the costs compared with autologous CAR T cell products or fresh allogeneic NK cell products (12, 23). We previously described the production of tumor-reactive or activated-by-cytokine killer (TRACK) NK cells expanded from umbilical cord blood (UCB) NK cells, transduced with a retroviral vector encoding soluble IL-15 (sIL15), which were further cytokine-activated to induce PD-L1 expression and then cryopreserved (24). The activated sIL15_TRACK NK cells have high expression of the activating NK cell receptors NKG2D, DNAM-1, and NKp30. Our preclinical results showed that thawed sIL15_TRACK NK cells were sustained in vitro by sIL15 in an autocrine and/or paracrine fashion for up to 42 days compared with the nontransduced NK cells that were alive for less than 9 days (24). Furthermore, sIL15_TRACK NK cells significantly improved cytotoxicity against NSCLC tumor cell lines in vitro when compared with nontransduced NK cells, nonactivated NK cells, PD-L1^+^ NK cells lacking sIL15 expression, or NK cells expressing sIL15 without further cytokine activation. Repeated i.v. injection of thawed sIL15_TRACK NK cells into NSG mice engrafted with a human NSCLC cell line significantly slowed tumor growth and improved survival when compared with all control groups. The addition of the anti–PD-L1 antibody atezolizumab further improved control of the NSCLC cell line growth by sIL15_TRACK NK cells in vivo. Moreover, a dose-dependent efficacy was achieved for sIL15_TRACK NK cells without substantial toxicity in animal models (24).

These preclinical studies suggested that unmatched, allogeneic, off-the-shelf sIL15_TRACK NK cells could be safely administered and possibly provide meaningful clinical benefit in patients with advanced NSCLC. To further evaluate the safety, toxicity, and potential efficacy of the sIL15_TRACK NK cell therapy, we initiated a first-in-human phase I clinical trial and herein provide an interim report on the first 6 patients with advanced, previously treated NSCLC who had relapsed from or were refractory to chemotherapy, refractory to immune checkpoint inhibition, and experiencing progression at the time of enrollment (25). The primary objective of this trial is to determine the safety and tolerability of the sIL15_TRACK NK cell therapy, while also investigating pharmacokinetics, evidence of trafficking, antitumor activity, and immune response. Additionally, we have demonstrated the feasibility of manufacturing and cryopreserving sIL15_TRACK NK cells under good manufacturing practice conditions, ensuring that the therapy can be scaled up for expanded clinical trial application in NSCLC and possibly other diseases.

## Results

Six patients with NSCLC have been enrolled to date in this first-in-human dose-escalation clinical trial of sIL15_TRACK NK cells at dose level 1 (DL1) (3 patients) and DL2 (3 patients). The patients’ clinical characteristics and tumor genomic profiles are shown in Table 1. Patients had a median age of 63.5 years (range, 45–80 years) and previously received a median of 3 chemotherapy and immune checkpoint inhibitor regimens (range, 2–6).

**Figure 1:**
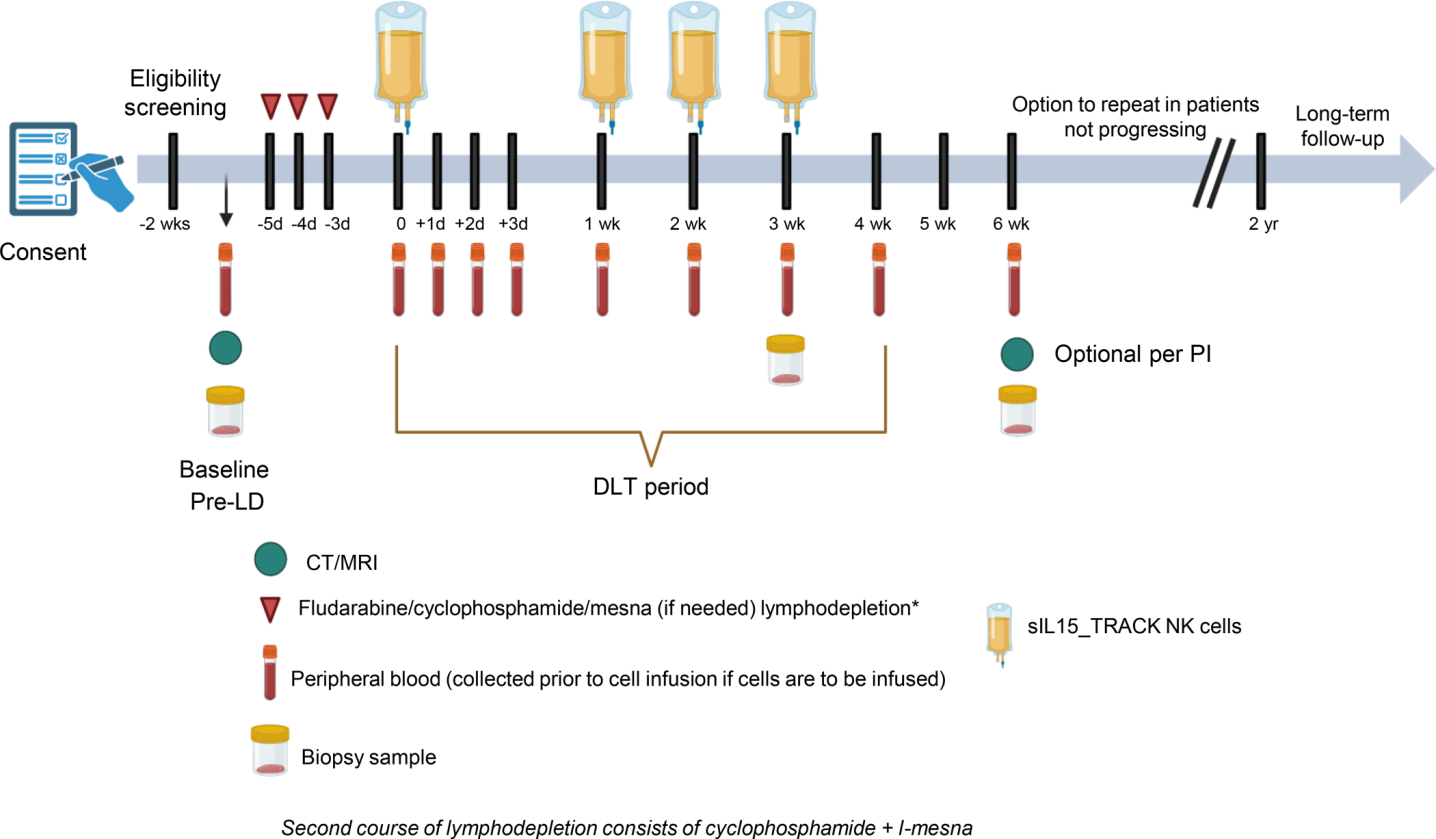
Protocol schema for first-in-human phase I trial of sIL15_TRACK NK cells in NSCLC (NCT05334329). LD, lymphodepletion; PI, principal investigator.

All patients received 3 days of the lymphodepletion regimen (LDR) starting 5 days prior to the first infusion of sIL15_TRACK NK cells (Figure 1). Following the LDR, patients received 4 weekly i.v. infusions of sIL15_TRACK NK cells that had been cryopreserved for ≥4 months, thawed at the bedside, and infused. Three patients were infused with 1.5 × 10^6^ transduced sIL15_TRACK NK cells/kg (DL1), and 3 patients received 4.0 × 10^6^ transduced sIL15_TRACK NK cells/kg (DL2) (Table 2). The LDR was well tolerated, but 4 patients did have grade 3 or grade 4 cytopenia related to the LDR. Infusion-related reactions, evidence of cytokine release syndrome, or CNS toxicity following administration of the sIL15_TRACK NK cells were not observed. The most common grade 1–2 toxicities of the therapy included fatigue, nausea, and diarrhea (Table 3). One patient (patient 005) experienced a transient shortness of breath immediately following his seventh infusion of sIL15_TRACK NK cells at a dose of 4.0 × 10^6^ transduced cells/kg (second dose level). A chest x-ray was obtained that showed a transient pulmonary infiltrate, most consistent with trafficking of the sIL15_TRACK NK cells to the lung (Supplemental Figure 1; supplemental material available online with this article; https://doi.org/10.1172/jci.insight.186890DS1). Both the shortness of breath and the infiltrate resolved without fever or further intervention within 7 days.

**Figure 2:**
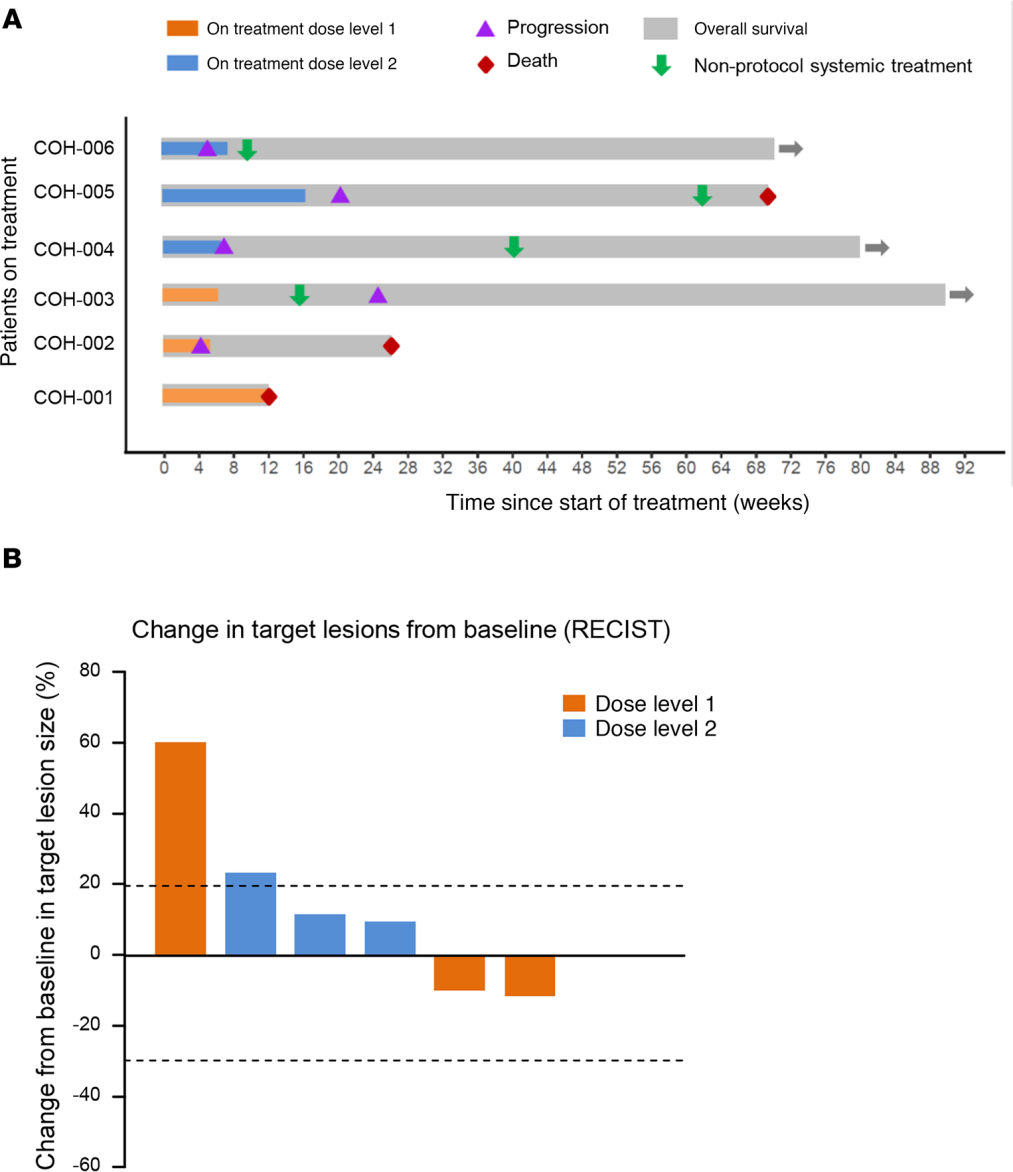
Depiction of patient outcomes. (**A**) Swimmer plot for all 6 patients. Three patients (patients 001, 003, and 005) exhibited stable disease prior to progression. One patient (patient 001) died unexpectedly of a cardiac event with a diagnosis of COVID while on the second treatment cycle without progression. Three patients (patients 002, 004, and 006) exhibited no stabilization of disease. The swimmer plot also shows systemic palliative treatment following treatment with sIL15_TRACK NK cells. Three patients remained alive after 70, 80, and 90 weeks. (**B**) Waterfall plot for all 6 patients showing change in target lesions based on Response Evaluation Criteria in Solid Tumors (RECIST). The tumor volume of the target lesions in patient 001 following 1 cycle of therapy with sIL15_TRACK NK cells was reduced by approximately 12% (Supplemental Figure 2).

All 6 patients were able to complete the planned 4 weekly infusions of sIL15_TRACK NK cells, with 1 patient (patient 005) receiving an additional course per protocol, consisting of 3 weekly sIL15_TRACK NK cell infusions following lymphodepletion consisting of cyclophosphamide alone. All 6 patients were evaluated for response with posttreatment CT imaging 2 weeks after the final infusion. Of these, 3 (50%) were determined to have stable disease at week 6 after the initiation of therapy, and 3 (50%) had progressive disease within the early follow-up period of 6 weeks. Among patients with stable disease, 1 (patient 001) died during the second treatment cycle without evidence of disease progression from a cardiac event and an active COVID infection; both were felt to be unrelated to the study. A swimmer plot (Figure 2A) and a waterfall plot (Figure 2B) show the clinical course of study participants, including subsequent palliative therapy provided to 4 of the 6 patients. Two of the patients in the first cohort showed a modest (10%–12%) decline in the target lesion tumor volume at week 6 (Figure 2B and Supplemental Figure 2).

**Figure 3:**
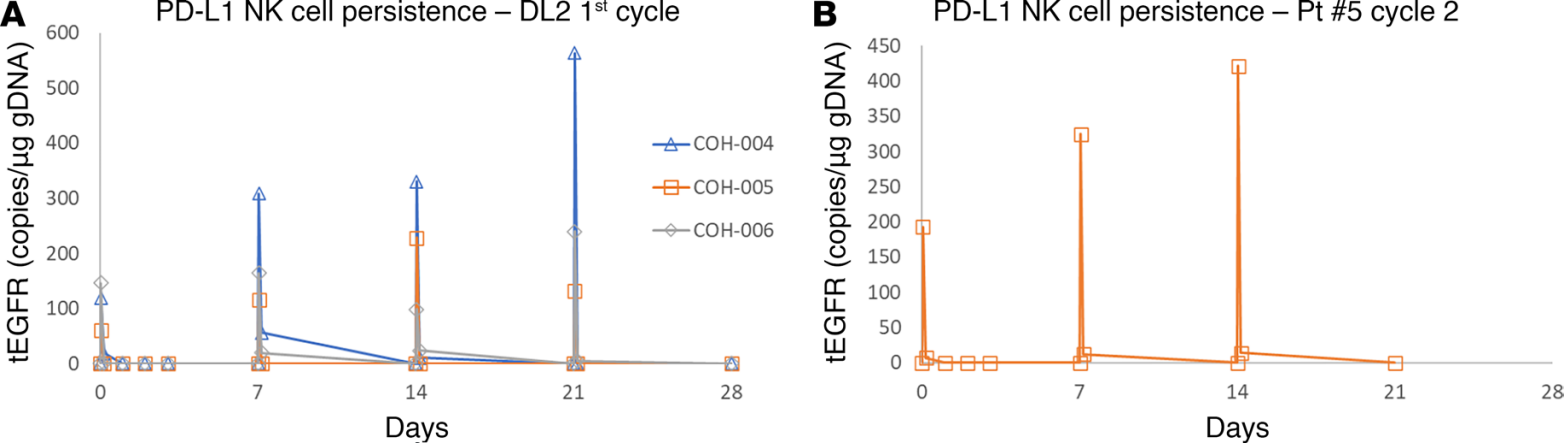
ddPCR assessment of sIL15_TRACK NK cells in vivo. (**A**) ddPCR assessment of sIL15_TRACK NK cells present in the sera of 3 patients, as measured at 0 hour (before infusion), 1 hour (shown), and 4 hours (data not shown) following the infusion of sIL15_TRACK NK cells on days 0, 7, 14, and 21 during the first cycle of therapy at DL2. Despite weekly equivalent dosing, at times we noted a progressive albeit modest increase in the 1-hour signal from day 0 to day 21. (**B**) ddPCR assessment of sIL15_TRACK NK cells present in the serum of patient 005, as measured at 0 hour (before infusion), 1 hour (shown), and 4 hours (data not shown) following the infusion of sIL15_TRACK NK cells on days 0, 7, and 14 during the second cycle of therapy at DL2. Again, despite weekly equivalent dosing, a progressive albeit modest increase in the 1-hour signal from day 0 to day 14 is noted, suggesting somewhat of a cumulative effect of the dosing.

Using droplet digital PCR (ddPCR), we evaluated sIL15_TRACK NK cell persistence in the blood of patients following sIL15_TRACK NK cell infusion at DL2 at time 0 (before infusion), 1 hour, and 4 hours on days 0, 7, 14, and 21 during the first treatment cycle (for patients 004, 005, and 006) and on days 0, 7, and 14 during the second treatment cycle (limited to patient 005). Transient elevation of sIL15_TRACK NK cells was detected in blood samples drawn in the first hour after the sIL15_TRACK NK cell infusion among the 3 patients treated on days 0, 7, 14, and 21 during the first treatment cycle and for the only patient (005) treated on days 0, 7, and 14 during the second treatment cycle but was gone from the circulation by within 4 hours of each infusion (Figure 3). However, despite weekly equivalent dosing, at times we noted a progressive albeit modest increase in the 1-hour signal from day 0 to day 21 (Figure 3A) or from day 0 to 14 (Figure 3B), suggesting somewhat of a cumulative effect of the dosing.

Plasma cytokines and chemokines were serially assessed in all 6 participants throughout the 28-day dose-limiting toxicity (DLT) period. The data displayed a very wide inter- and intraparticipant variability, and there were no systematic changes observed in the plasma concentrations of any of the circulating markers in response to sIL15_TRACK NK cell treatment. For example, baseline plasma IL-15 levels ranged from undetectable to being above the quantifiable range of the assay, and the concentrations did not increase after either single or multiple doses.

**Figure 4:**
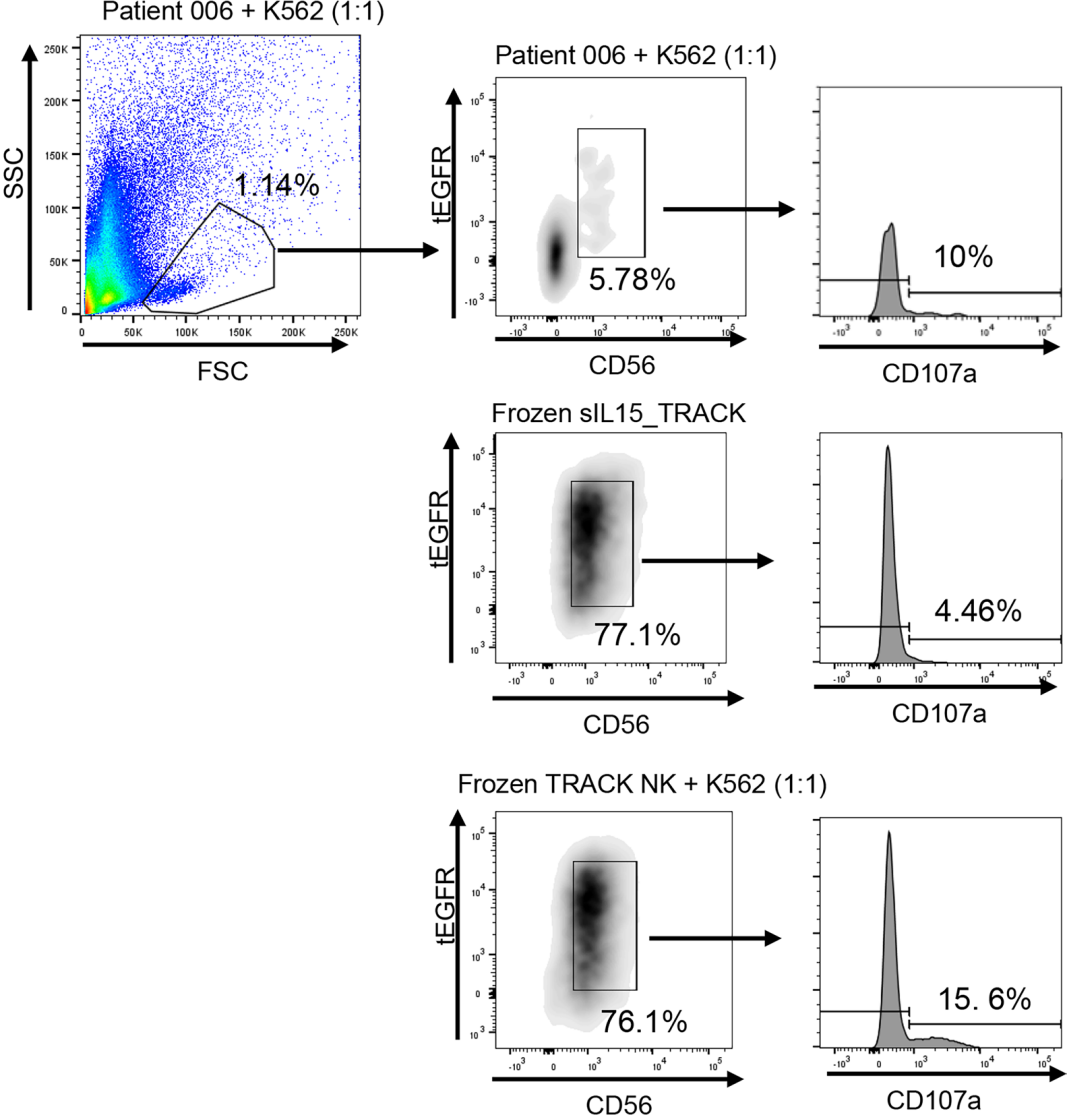
Flow cytometric assessment of patient lung tumor skinny needle biopsy. The biopsy from patient 006 was collected 24 hours following the fourth (final) infusion of sIL15_TRACK NK cells and was digested to single-cell suspension using collagenase I and DNase I. Cells cocultured with K562 tumor target cells for 4 hours were also stained with antibodies against CD56 and tEGFR and assessed by flow cytometry to identify tCD56^+^tEGFR^+^ sIL15_TRACK NK cells within the lymphocyte gate. The activation and degranulation of sIL15_TRACK NK cells in the presence of the K562 tumor target cells were measured by CD107a. Frozen and thawed sIL15_TRACK NK cells cocultured without (middle) or with (bottom) K562 tumor target cells were stained as negative and positive controls for CD107a, respectively.

**Figure 5:**
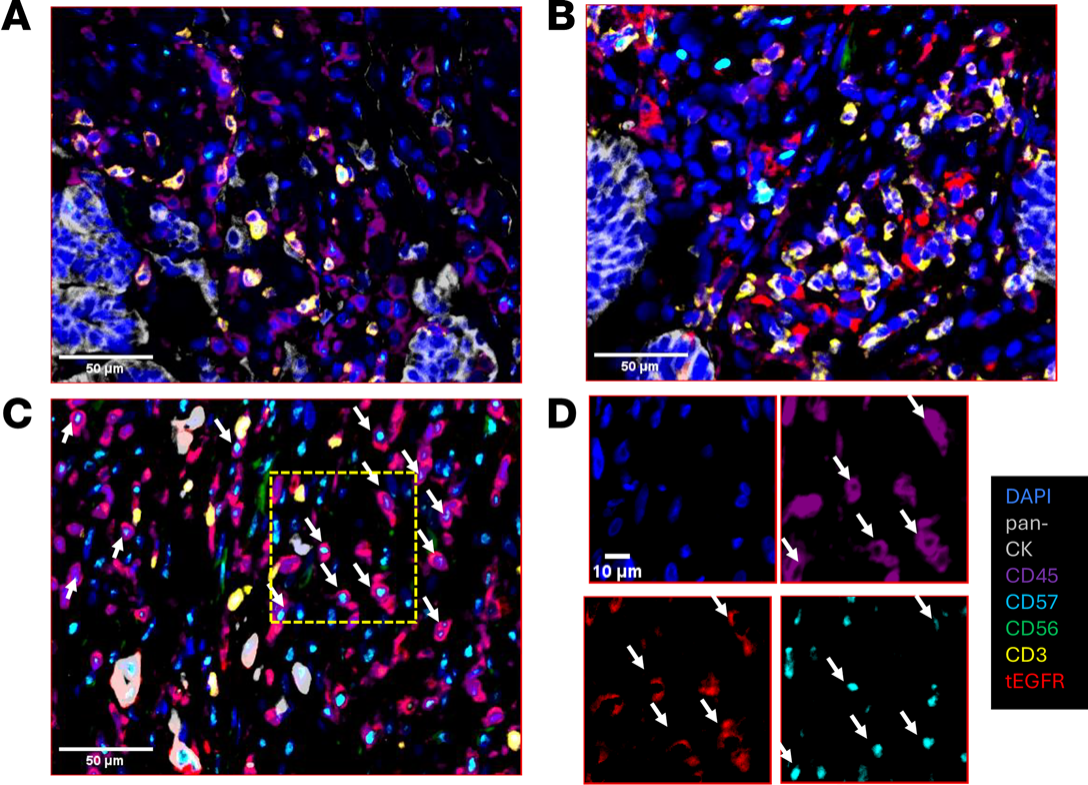
Immunofluorescence histochemical staining of patient lung tumor biopsy. Utilizing the multicolor immunofluorescence histochemical staining, we examined lung tumor tissue from patient 006 on 3 occasions using DAPI (blue) for nuclear staining, pan-CK (gray) for lung parenchyma, CD45 (purple) for lymphocytes, CD57 (cyan) and CD56 (green) for NK cells, CD3 (yellow) for T cells, and EGFR (red) combined with CD56 or CD57 to identify NK cells transduced with the tEGFR-sIL15 construct. (**A**) FFPE tissue from a pretreatment tumor biopsy from patient 006 shows the presence of lung parenchyma (DAPI + pan-CK^+^) but the absence of tEGFR^+^ (red) transduced CD57^+^ or CD56^+^ NK cells. (**B**) FFPE tissue from the skinny needle tumor biopsy collected 24 hours following the fourth and final infusion of sIL15_TRACK NK cells without observable of tEGFR^+^ (red) transduced CD57^+^ (cyan) or CD56^+^ (green) cells. (**C**) FFPE tissue from an excisional tumor biopsy collected 7 days following the fourth and final infusion of sIL15_TRACK NK cells shows sIL15_TRACK NK cells identified in DAPI^+^CD45^+^ cells by coexpression of tEGFR (red) with CD57 (cyan), as marked by white arrows. (**D**) Selected individual markers of the boxed region shown in **C** are shown. Scale bars: 10 μm (**A**–**C**); 10 μm (**D**).

Patients also underwent CT- or ultrasound-guided skinny needle biopsies of lung tumor tissue within 7 days following the fourth infusion of sIL15_TRACK NK cells during the first treatment cycle, at a time when these cells were no longer detected in the circulation. Flow cytometric plots assessing forward scatter versus side scatter of biopsied material from patients 002, 004, and 005, who underwent skinny needle biopsy, showed very few (<0.5%) cells collected in the lymphocyte gate where sIL15_TRACK NK cells would reside and, consequently, none were detected (Supplemental Figure 3). While skinny need biopsy in the lung is notoriously bad for adequate sampling of tumor and immune cells, it is well known from extensive mouse studies, including our own published work (26), and human studies (11, 12), that i.v. injected NK cells first traffic to lung and lung tumors. Patient 006 also underwent a skinny needle biopsy of tumor 1 day after the fourth infusion of sIL15_TRACK NK cells during the first treatment cycle, and it contained sufficient cells, both within the lymphocyte gate and outside of the gate where macrophages, lung parenchyma, and tumor cells are found. Dual staining for CD56 (on all NK cells) and an antibody that selectively detects the truncated form of EGFR (tEGFR, a surface marker transduced within the sIL15 construct to identify transduced NK cells) was performed followed by flow cytometric analysis, demonstrating a relative abundance of sIL15_TRACK NK cells in the biopsy from patient 006 (Figure 4). As an assessment of function, the same sIL15_TRACK NK cells that were isolated from the lung skinny needle biopsy of patient 006 within 24 hours following the final infusion were cocultured for 4 hours in vitro alongside the standard NK cell target tumor cell line K562, demonstrating cytolytic granule exocytosis by CD107a staining (Figure 4). This same specimen from patient 006 also underwent multicolor immunofluorescence histochemical staining, in which DAPI detected cell nuclei, pan cytokeratin 20 mAb detected lung parenchyma, CD45 mAb detected lymphocytes, CD56 or CD57 mAbs detected NK cells, CD3 mAb detected T cells, and an EGFR mAb combined with CD56 or CD57 mAbs selectively detected the tEFGR-transduced NK cells. When compared with a pretreatment tumor biopsy from patient 006 (Figure 5A), the patient 006 sample obtained 24 hours after the final (fourth) infusion of sIL15_TRACK showed evidence of NK cells and T cells along with evidence of transduction with the vector containing both tEGFR and sIL15 (Figure 5B).

We next obtained an excisional biopsy of tumor from patient 006 performed 7 days following the final infusion of sIL15_TRACK NK cells. Utilizing the same multicolor immunofluorescence histochemical staining we obtained definitive visual evidence of sIL15_TRACK NK cells trafficking into the lung TME, identified by the coexpression of CD57 with tEGFR (Figure 5, C and D) (24). The CD56 stain was optimized for detection of CD56^bright^ NK cells, and the cells detected trafficking into the tissues were the most mature NK cells, i.e., CD56^dim^ CD57^+^ NK cells. Therefore, we see more intense staining for the other NK cell marker, CD57 (cyan, Figure 5, C and D). Thus, while sIL15_TRACK NK cells were no longer detectable by ddPCR in the peripheral circulation within 4 hours of the infusion (Figure 3), an abundance of sIL15_TRACK NK cells were detectable within the TME on day 28 (7 days following the last infusion), demonstrating successful trafficking to the tumor site as well as their sustainability in vivo.

Because we were able to document cytotoxicity of the sIL15_TRACK NK cells within 24 hours after the infusion, and document the presence of sIL15_TRACK NK cells at the tumor site 7 days after the final infusion, we assessed the sIL15_TRACK NK cells for the expression of 3 activating receptors, namely NKG2D, DNAM-1, and NKp30, among other antigens and the NSCLC tumor biopsy for its expression of the cognate ligands to those 3 receptors. The phenotype of thawed sIL15_TRACK NK cells shown in Supplemental Figure 4 was assessed immediately after thawing and prior to infusion as well as following a 48-hour culture at 37°C to mimic in vivo conditions. Notably, all 3 NK cell–activating receptors (NKG2D, DNAM-1, and NKp30) show high surface density expression after 48 hours in culture, as do CD16 and NKp44. sIL15_TRACK NK cells also show high surface density expression of CD69 and PD-L1, consistent with a heightened state of NK cell activation, along with modest surface expression of NKG2C (Supplemental Figure 4) (9).

**Figure 6:**
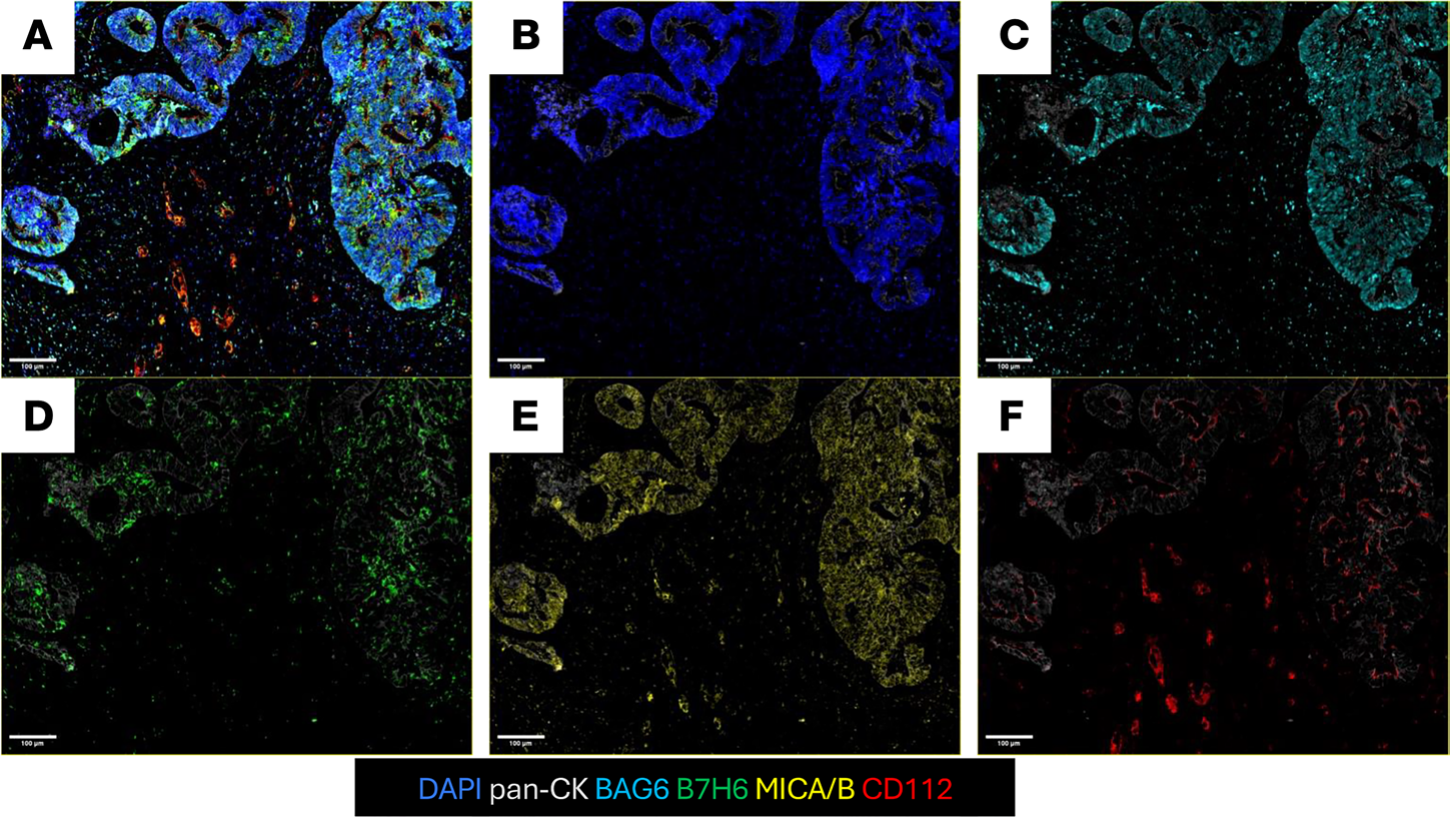
NK cell–activating receptor ligands expressed on tissue from patients with NSCLC. An excisional tumor biopsy specimen from patient 006 was assessed for the presence of the cognate ligands to NKp30 (i.e., BAG6 or B7H6), NKG2D (i.e., MICA/B), and DNAM-1 (i.e., CD112). Tissues were stained by multiplex immunofluorescence for DAPI (blue), pan-cytokeratin (CK, gray), BAG6 (cyan) B7H6 (green), MICA/B (yellow), and CD112 (red). Representative images are shown from posttreatment FFPE tumor tissue as a composite image (**A**), and as stained with pan-CK and DAPI (**B**), pan-CK and BAG6 (**C**), pan-CK and B7H6 (**D**), pan-CK and MICA/B (**E**), and pan-CK and CD112 (**F**). Scale bars: 100 μm.

We next assessed the NSCLC tumor biopsy from patient 006 for expression of the cognate ligands for NKGD2 (i.e., MICA/B), DNAM-1 (i.e., CD112), and NKp30 (i.e., B7H6 and BAG6). As can be seen in the multicolor immunofluorescence histochemical staining (Figure 6), ligands to all 3 of the activating NK cell receptors of NKG2D, DNAM-1, and NKp30 can be found within the lung tumor tissue obtained from patient 006.

Collectively, our correlative data suggest that exogenously administered unmatched, allogeneic, cryopreserved, off-the-shelf sIL15_TRACK NK cells are safe to infuse and traffic from peripheral blood to the tumor site where they can be found for an extended period beyond their detection in the peripheral circulation. Additionally, these cells express activating NK cell receptors whose cognate ligands are expressed on the tumor biopsies from patients with NSCLC and retain cytotoxic activity.

## Discussion

In this interim report of study NCT05334329, we assessed the safety, tolerability, and potential efficacy of unmatched, allogeneic off-the-shelf sIL15_TRACK NK cell infusions in the first 6 patients with NSCLC that were refractory to or relapsed after chemotherapy and immune checkpoint therapy and demonstrated progressive disease at the time of study enrollment. We performed several correlative studies to assess in vivo sIL15_TRACK NK cell pharmacokinetics, survival, trafficking, and biologic activity where patient material was available.

IL-15 is both a survival and activation factor for NK cells in vivo (27–29). It is produced by antigen-presenting cells, such as DCs, which express the IL-15 receptor α (Ra) chain that binds IL-15 with high affinity. IL15Ra presents IL-15 in *trans* to NK cells expressing the heterodimeric IL15Rb and common g chains (30, 31). Experimental evidence has demonstrated that NK cell homeostasis depends on the amount of endogenous IL-15 that is available. When endogenous IL-15 supplies increase, the number of total NK cells increases (32), and when IL-15 is absent or limited, the number of NK cells drops precipitously (33, 34). This suggests that there is no excess endogenous IL-15 in humans to sustain multiple infusions of large numbers of ex vivo–generated NK cells that are not producing their own IL-15. Therefore, engineering the TRACK NK cell to produce its own sIL15 allows the exogenously administered effector cells to be sustained and activated once infused and to do so independent of the patient’s endogenous IL-15 stores. Furthermore, it has been shown that exogenous IL-15 can enhance T cell responsiveness to checkpoint inhibition (35). We had previously demonstrated measurable amounts of sIL15 when sIL15_TRACK NK cells were incubated in culture (24). Thus, the local production of sIL15 within the TME by the sIL15_TRACK NK cells may further activate local T cell populations when anti–PD-L1 mAb is administered along with sIL15_TRACK NK cells following the achievement of an optimal biologic dose (OBD) of sIL15_TRACK NK cells as is planned for the second cohort of this study (Table 2).

We have also previously provided preclinical evidence that activated, human sIL15_CAR NK cells traffic directly from the venous circulation to the lung where they collect transiently before traveling onto the liver (26). This likely results from both the recognition of pulmonary endothelial adhesion molecules by receptors expressed on activated NK cells and the transient physical barrier created by the pulmonary capillary system. In the current study, we add more definitive data to support activated NK cells trafficking to the lungs. We provided radiographic evidence of a transient infiltrate in one patient (patient 005) 5 days following infusion of sIL15_TRACK NK cells (Supplemental Figure 1) and biopsy-proven evidence of the sIL15_TRACK NK cells within the lung in a second patient (patient 006), the latter obtained 7 days after the final infusion of sIL15_TRACK NK cells and well after they were no longer detectable in the circulation (Figure 5, C and D). Thus, in contrast to the challenges seen with CAR T cells in the setting of solid tumor thus far (36), we have demonstrated that our gene-modified sIL15_TRACK NK cells can home to the primary site of disease in patients with NSCLC and can do so without expression of a CAR.

The importance of PD-L1 expression on the surface of the sIL15_TRACK NK cell has yet to be tested clinically but will be examined in the second half of this study. As noted above, Dong et al. observed upregulation of PD-L1 on the surface of a subset NK cells that recognize their tumor target both in vitro and in vivo (9). The PD-L1^+^ NK cell subset is functionally more active when compared with the PD-L1^–^ subset. Additionally, the binding of the anti–PD-L1 mAb atezolizumab to the PD-L1^+^ NK cell further activates the cell via a p38/NF-κB pathway, resulting in an increase in secretion of cytotoxic granules and IFN-γ as well as further upregulation of PD-L1 via a positive feedback loop (9). By incubating the transduced sIL15_NK cells with a combination of NK cell–activating cytokines ex vivo, as occurs in the final step of the sIL15_TRACK NK cell manufacturing process immediately prior to cryopreservation, we can vastly upregulate PD-L1 on the surface of the sIL15_NK cell, and the resultant sIL15_TRACK NK cell retains PD-L1 expression following the thaw and infusion into the patient (Supplemental Figure 4). As the administration of atezolizumab has shown significant activity as a single agent in NSCLC by blocking the PD-1–PD-L1 pathway (37), we would expect that its antitumor, immune checkpoint inhibition could be enhanced further via its direct binding to and activation of the PD-L1^+^ sIL15_TRACK NK cells as noted above. Once the OBD of sIL15_TRACK NK cells to be infused is determined in our phase I study, we will administer the OBD in the presence of atezolizumab for the remaining 6–12 patients in this ongoing study (Figure 1).

In addition to trafficking to the site of disease, NK cells must recognize tumor-associated antigens and retain their cytotoxic function. sIL15_TRACK NK cells in our study are not engineered to express a CAR but do show high surface density expression of 3 activating NK cell receptors: NKG2D, whose ligands are MICA/B; DNAM-1, whose ligands are CD155/CD112; and NKp30, whose ligands are B7-H6/BAG6 (18–21) (Supplemental Figure 4). Analysis of MICA expression in 222 NSCLC cases revealed that 98.2% of the lung tumors showed positive staining for MICA, with nearly half demonstrating high surface density expression of the ligand (38). CD155 expression was found to be positive in 37 (38.5%) of 96 patients with completely resected stage I adenocarcinoma of the lung. CD155 positivity was associated with aggressive tumor behavior and was a significant predictor of a poor prognosis (39). B7-H6 or BAG6, the ligands for NKp30, are found in slightly less than 10% of NSCLC cases (15). Thus, there is very high probability that any one NSCLC will express at least one of these ligands, thereby allowing for tumor cell recognition by sIL15_TRACK NK cells in addition to trafficking. Indeed, multicolor immunofluorescence histochemical staining of 1 excisional tumor biopsy from 1 patient with NSCLC in our study demonstrated the expression of all 3 ligands within the tumor (Figure 6). However, this receptor-ligand recognition could be different from the interaction between a CAR and a targeted antigen, where CD3ζ and a costimulatory molecule are usually included in the construct. Additionally, as noted earlier, relapsed NSCLC often loses MHC class I expression, thereby increasing its susceptibility to NK cell killing, even in the absence of such ligands. Finally, we demonstrated that the sIL15_TRACK NK cells obtained from a lung needle biopsy within 24 hours of the final infusion retained cytotoxic activity against a standard tumor cell target, despite the infused product being cryopreserved for over 4 months and within the patient for approximately 24 hours. These properties of trafficking to the TME, recognition of tumor-associated ligands with multiple endogenous activating NK cell receptors, and retention of cytotoxic function likely explain the intriguing results of improved overall survival obtained in the randomized trial of patients with advanced NSCLC who received anti-PD-1 mAb therapy with infusions of freshly activated unmatched NK cells (i.e., no cryopreservation), compared with those who received only anti-PD-1 mAb (22). Furthermore, since sIL15 allows for sustained survival and activation in vivo (24, 40), the addition of sIL15 to the TRACK NK cells may allow for an antitumor response at the OBD beyond the short stabilization of disease that we observed in 50% of patients enrolled on this study thus far, assuming cytotoxic activity of the product can be maintained throughout time of cryopreservation. While the observed achievement of stable disease in 3 of the 6 patients following the sIL15_TRACK NK cell infusions may be relevant considering the advanced and progressive stage of the disease at the time of treatment in all 6 patients, the palliative treatments consisting of localized radiation and repeat dosing of prior chemotherapy weeks or months after achieving stable disease preclude us from drawing any conclusions about the longer-term efficacy of the regimen. It is intriguing that 2 of the 6 patients experienced a slight (≤12%) reduction in tumor size prior to any further treatment (Figure 2B and Supplemental Figure 2). However, the absence of a complete or partial response in these first 6 patients supports the need for further optimization of this therapy. Nonetheless, the surface expression of activating receptors on the sIL15_TRACK NK cells as well as the broad expression of their cognate ligands on other malignancies would suggest that this form of immune therapy, in the absence of a CAR, may have broader applicability in a multitude of liquid and solid tumors that also express these ligands.

Finally, should this form of cellular therapy prove to be efficacious in advanced NSCLC, we must face the challenge of developing a product that both is accessible to the millions of patients with lung cancer and can be delivered at an affordable cost. While activity in both lymphoma (23) and NSCLC (22) has been reported using unmatched allogeneic, activated NK cells derived from UCB and peripheral blood, respectively, and both studies have been reported as very safe, both products have been freshly isolated and infused, which limits simultaneous widespread distribution. The sIL15_TRACK NK cell product described in this report that is derived from UCB NK cells is expanded, transduced, undergoes further expansion, and final cytokine activation followed by viable cryopreservation, allowing for the utilization of the same product at a multitude of sites and on a multitude of dates whenever the patient is ready to receive the product, thereby lowering production costs and improving the widespread access.

In summary, we provide early clinical data documenting the safety and tolerability of an allogeneic, unmatched, activated, engineered, and cryopreserved off-the-shelf NK cell product, sIL15_TRACK NK cells, delivered as an outpatient infusion, trafficking to the site of the tumor while retaining some cytotoxic function in the lung after the final infusion in the setting of advanced and progressive NSCLC. These data, and those published previously using fresh unmatched allogeneic NK cells in advanced NSCLC (22), as well as the planned addition of atezolizumab to the OBD of this regimen as noted in the protocol, suggest that continued exploration of the sIL15_TRACK NK cell therapy in such patients is warranted.

## Methods

### Sex as a biological variable.

Both male and female patients have been enrolled in this study.

### Study design and participants.

This is an interim evaluation of a single-center, first-in-human, ongoing clinical trial in which adults with advanced, metastatic, or recurrent NSCLC (having disease progression after treatment with chemotherapy and/or PD-1 and/or PD-L1 immune checkpoint inhibitors) received UCB-expanded and activated NK cells that have been genetically modified to constitutively express sIL15 (sIL15_TRACK NK cells or COH06) as monotherapy (Figure 1). Eligibility criteria included the following: (a) age ≥18 years; (b) histologically confirmed stage IV or recurrent NSCLC with radiographically demonstrable tumor progression after treatment with a PD-1 or PD-L1 immune checkpoint inhibitor; (c) performance status of 0 or 1 in the Eastern Cooperative Oncology Group scale; (d) preserved organ function and resolution of any prior treatment-related toxicities to ≤ grade 1; (e) cardiac ejection fraction ≥50% and no clinically relevant electrocardiogram findings; and (f) willingness to undergo posttreatment tumor biopsies. The presence of active brain metastases was a criterion for exclusion unless they were treated and stable on subsequent magnetic resonance scans. Patients with known positive serology for HIV or history of severe (≥ grade 3) immune-related adverse events during prior PD-1 or PD-L1 inhibitor treatment were also excluded.

sIL15_TRACK NK cells were i.v. administered following lymphodepleting chemotherapy. We utilized a standard outpatient LDR in which i.v. 30 mg/m^2^ fludarabine, 300 mg/m^2^ cyclophosphamide, and 300 mg/m^2^ mesna (see note below) were administered for a total of 3 days followed by 2 days of rest (23). Patients who were treated with a second cycle of sIL15_TRACK NK cells received only cyclophosphamide (with or without 300 mg/m^2^ mesna). Note: Given the lower doses of the LDR being used in this study, mesna was not given for the first course of treatment but was made available for the second cycle of treatment if toxicity was observed. Initiation of LDR commenced 5 days prior to the first infusion of sIL15_TRACK NK cells. The LDR doses were based on actual body weight, unless the actual body weight exceeded 120% of the ideal body weight, in which case the adjusted body weight was used.

The starting sIL15_TRACK NK cell dose (DL1) was 1.5 × 10^6^ transduced cells per kg, given weekly for 4 weeks to 3 patients, followed by DL2, 4.0 × 10^6^ transduced cells per kg, given weekly for 4 weeks to 3 patients. Subsequent dose levels that are planned include DL3, 1.2 × 10^7^ transduced cells per kg, and DL4, 1.2 × 10^7^ transduced cells per kg (or the maximum safe dose level if below or above DL3) plus atezolizumab (840 mg i.v.), every 2 weeks, which if safe will be followed by DL5, 2.0 × 10^7^ transduced cells per kg plus atezolizumab (840 mg i.v.) every 2 weeks (Table 2). At each dose level patients are enrolled in cohorts of 3, with the first 2 dose levels completed in this report. There was no intrapatient dose escalation. Antitumor responses, as evaluated by CT or MRI scans, were conducted at week 6 (i.e., 2 weeks after the fourth sIL15_TRACK NK cells infusion). Patients demonstrating antitumor response or disease stabilization and who tolerated the first 4 infusions without DLT were offered the opportunity to receive a second cycle if the treating physician felt that treatment continuation was appropriate. Toxicity was graded according to two grading systems: (a) the National Cancer Institute Common Terminology Criteria for Adverse Events, version 5.0, and (b) ASTCT Consensus Grading for Cytokine Release Syndrome (CRS) and neurotoxicity associated with immune effector cells (41).

DLT was defined as any grade 3 or higher nonhematologic toxicity designated as probably or definitely related (level of attribution) to sIL15_TRACK NK cells and occurring within 28 days of the first sIL15_TRACK NK cell infusion with the exception of rapidly reversible (within 7 days) nausea/vomiting, diarrhea, and skin rash. For immune-mediated toxicities the following will be considered DLTs: (a) any grade 3 CRS that does not resolve to ≤ grade 1 within 7 days; (b) any grade 4 or higher CRS; (c) any grade 2 or higher neurotoxicity that does not resolve to grade 1 within 72 hours; (d) any grade 3 autoimmune, hypersensitivity, and acute graft-versus-host reactions; and (e) any grade 4 or higher hematological toxicity that does not improve to ≤ grade 2 within 14 days, despite standard interventions and supportive measures, and is designated as at least possibly related to sIL15_TRACK NK cells. Patients were to be followed for disease progression and survival for up to 2 years. In addition, research participants will continue with annual evaluations for a minimum of 15 years for replication competent retrovirus, as part of a City of Hope long-term follow-up protocol, in accordance with the FDA guidance “Gene Therapy Clinical Trials – Observing Subjects for Adverse Events” (42).

Pretreatment archival lung cancer tissue was obtained, or a pretreatment biopsy was performed, if archival material had been depleted. A tumor biopsy was also performed within 2 weeks of the fourth infusion of sIL15_TRACK NK cells.

The primary objectives were (a) to assess the safety and determine the optimal biological dose (OBD) of sIL15_TRACK NK cells and (b) to assess the cellular kinetics of sIL15_TRACK NK cells through the detection and measurement of persistence in the peripheral blood or lung tissues.

Secondary objectives included (a) estimating the overall response (complete response plus partial response) and disease control (complete response plus partial response plus stable disease) rates, including duration, and (b) estimating the progression-free survival and overall survival rate at 6 months and 1 year following the first sIL15_TRACK NK cell infusion. Correlative Study Objectives included assessment on tumor biopsies for the presence and activation status of sIL15_TRACK NK cells via flow cytometry, persistence by digital droplet PCR (ddPCR), cytokine analysis, immunohistochemical staining and assessment of T cell activation by flow cytometry, and cytokine analysis.

### Manufacturing process.

sIL15_TRACK NK cells were manufactured as previously reported under good manufacturing practice conditions (24). Briefly, UCB was obtained from StemCyte and enriched for UCB NK cells. The enriched UCB NK cells were cryopreserved in CryoStor CS5. Once thawed, the UCB NK cells were cocultured with irradiated K562 feeder cells expressing membrane-bound IL-21 and CD-137L (gifted by CytoImmune Therapeutics), along with exogenous IL-2. On day 7, the expanded NK cells were transduced with the retroviral vector (RRV_sIL15_tEGFR) carrying the human IL15 gene and the tEGFR. Following transduction, the cells were further expanded in coculture with additional irradiated K562 feeder cells plus IL-2. On day 16, the cytokines IL-18 and IL-12 were added to the cell culture to upregulate endogenous expression of PD-L1 on the transduced NK cells expressing sIL15. On day 17, the sIL15_TRACK NK cells were harvested and cryopreserved.

### ddCR and quantitative ddPCR for NK cell persistence.

Genomic DNA (gDNA) was extracted from frozen aliquots of whole blood using the Maxwell automated nucleic acid extraction system (Promega). gDNA samples were diluted to a final concentration of 50 ng/μL, and 1 μL was used for each ddPCR reaction. The final reaction mix included PCR primer/probe sets for tEGFR (forward, 5′-TCCGGATTAGTCCAATTTGTTAAAG, reverse, 5′-TCTATGGCTCGTACTCTATAGGC, probe, 5′-ACAGGATATCAGTGGTCCAGGCTCT) and albumin (forward, TGAAACATACGTTCCCAAAGAGTTT; reverse, CTCTCCTFCTCAGAAAGTGTGCATAT, probe, TGCTGAAACATTCACCITCCATGCAGA) at final concentrations of 900 nM primer/250 nM probe, along with 10 μL ddPCR Supermix for Probes (Bio-Rad) and nuclease-free water to final volume of 20 μL. A no-template control with distilled water in place of gDNA was included with each run. Each 20 μL ddPCR reaction was loaded into a sample well of an 8-channel droplet generation cartridge (Bio-Rad), along with 70 μL QX200 droplet generation oil (Bio-Rad), and the cartridge was loaded into the QX200 Droplet Generator (Bio-Rad). A 40 μL aliquot of each resulting droplet emulsion was then transferred to a 96-well plate, which was sealed with pierceable foil (Bio-Rad) using a PX1 PCR Plate Sealer (Bio-Rad), and then the plate was transferred to a T100 Thermal Cycler (Bio-Rad) for amplification. Cycling conditions were as follows: 95°C for 10 minutes, 40 cycles of 94°C for 30 seconds, 60°C for 1 minute, and finally 98°C for 10 minutes. The temperature ramp rate was 2°C/second, and following amplification, plates were held at 4°C until they could be loaded into a QX200 Droplet Reader (Bio-Rad). Data analysis was performed using QX manager 1.2 standard edition software (Bio-Rad), including automatic Poisson correction, and the results were reported as copies of tEGFR per μg gDNA.

### Luminex multiplex immunoassay.

Samples were analyzed for 30 cytokines using the Human Cytokine Thirty-Plex Antibody Magnetic Bead Kit (Invitrogen) as per the manufacturer’s protocol. Briefly, Invitrogen’s multiplex bead solution was vortexed for 30 seconds, and 25 μL was added to each well of a flat-bottom 96 well microplate. Plasma samples were diluted 1:2 with assay diluent and loaded into the wells containing 50 μL incubation buffer. Cytokine standards were reconstituted with assay diluent, and serial dilutions of cytokine standards were prepared in parallel and added to the plate. Plates were incubated on a shaker in the dark at room temperature for 2 hours. The plate was then applied to a magnetic capture device and washed 3 times with 200 μL wash buffer. After the final wash, 200 μL of a biotinylated detection antibody mixture was added to each well, and the plate was incubated on a shaker for 1 hour. After washing again 3 times with 200 μL wash buffer, streptavidin-phycoerythrin (100 μL) was added to the wells. The plate was incubated on a plate shaker for another 30 minutes and washed 3 times, after which the beads were resuspended in 150 μL of wash buffer and shaken for 1 minute. Finally, the assay plate was transferred to a Flexmap 3D Luminex system (Luminex Corp) for analysis. Cytokine concentrations were calculated using Bio-Plex Manager 6.0 software with a 5 parameter curve-fitting algorithm applied for standard curve calculations for duplicate samples.

### Tumor cell preparation.

The NSCLC biopsy specimen was minced with blades into minute fragments and then transferred to a Miltenyi gentleMACS C tube. 5 mL RPMI dissociation buffer, which contains 0.5% BSA, 0.5 mM EDTA, 5 μL/mL DNase I, and 40 μL/mL Collagenase I, was added to the Miltenyi gentleMACS C tube. The tube was inserted into the gentleMACS, and the program h_tumor_02 was run. After tissue dissociation, the tube was placed on a MACSmix rotator in a 37°C incubator for 20 minutes to facilitate digestion. Before completing digestion, the program h_tumor_03 was run using the gentleMACS, followed by washing the tube with 10 mL PBS wash buffer (1% BSA and 0.5 mM EDTA). The digested tissue samples were transferred with the buffer to a 50 mL conical tube to remove the undigested tissues using a sterile 50 mL tube filter. The 50 mL conical tube was then centrifuged at 340*g* for 5 minutes, and the pellet was resuspended and collected for characteristic analysis by flow cytometry described below.

### Flow cytometry assessing phenotype and cytotoxic function.

Anti-human CD56 antibody (Biolegend, 318306, 5 μL/sample) and anti-human EGFR antibody (Biolegend, 352906, 5 μL/sample) were used for the flow cytometric analysis of sIL15_TRACK NK cells in the prepared samples. For assessing cytotoxic capacity of infused sIL15_TRACK NK cells, single cells isolated from a human lung tissue biopsy as aforementioned were cocultured with K562 cells for 4 hours, stained with an anti-human CD107a antibody (BD, 560949, 10 μL/sample), and then assessed for CD107 expression by flow cytometry. Frozen and thawed sIL15_TRACK NK cells cocultured without or with K562 cells were included as negative and positive controls, respectively. All flow cytometry data were collected using the Fortessa X-20 flow cytometer.

For surface density expression of other antigens, frozen sIL15_TRACK NK cells were either thawed for flow cytometry analysis without culturing or cultured in EL837 Serum-Free Medium (EliteCell Biomedical Corp.) with 5% human serum with low dose (100 IU/mL) IL-2 for 2 days before being collected for flow cytometry analysis. The following antibodies were used for the flow cytometric analysis: anti-human NKG2D (Miltenyi Biotec, 130-092-673, 10 μL/sample), anti-human NKG2C (Miltenyi Biotec, 130-103-635, 10 μL/sample), anti-human PD-L1 (Biolegend, 393608, 5 μL/sample), anti-human DNAM-1 (Biolegend, 338312, 5 μL/sample), anti-human CD69 (Beckman Coulter, IM1943U, 10 μL/sample), anti-human NKp30 (Biolegend, 325209, 5 μL/sample), anti-human NKp44 (Biolegend, 325116, 5 μL/sample), and anti-human CD16 (BD, 555406, 5 μL/sample).

All flow cytometry data were collected using the Fortessa X-20 flow cytometer (BD Biosciences).

### Immunohistochemistry.

FFPE tissue specimens were cut into 3–4 μm sections and baked onto glass slides. The FFPE slides were then deparaffinized in xylene and then rehydrated in decreasing ethanol concentration washes. Heat-induced antigen retrieval was performed using boiling AR9 buffer, 10x (pH 9) (Akoya Biosciences) in a microwave oven for 20 minutes. Blocking was performed for 10 minutes using Antibody Diluent with Background-Reducing Components (Agilent) to minimize nonspecific background staining. Primary antibodies were incubated for 1 hour on a shaker at room temperature followed by a 10-minute incubation of HRP-conjugated secondary antibody (Mouse HRP-Polymer, Biocare Medical). Immunofluorescence labeling of antibodies was achieved using the Opal 7-color fluorescence IHC Kit (Akoya Biosciences) at a 1:100 dilution for 10 minutes. Slides were serially stained with the microwave incubation acting to remove previous antibodies while simultaneously exposing the next epitope of interest. After staining the final marker, cell nuclei were stained with DAPI (Akoya Biosciences), and the slides were mounted with ProLong Gold Antifade Reagent (Thermo Fisher Scientific). For assessment of tumor infiltration of NK cells, primary antibodies were as follows: EGFR (clone 31G7, Novus), CD3 (clone LN10, Leica), CD56 (clone MRQ-42, Cell Marque), CD45 (clone D9M81, Cell Signaling Technologies), CD57 (clone HNK-1, Biolegend), and pan-cytokeratin (clone AE1/AE3, Dako). For assessment of NK cell receptor ligands, primary antibodies were as follows: BAT3/BAG6 (clone EPR9223, Abcam), B7H6 (clone B7H6/4821, Novus), MICA/B (clone 159207, Novus), CD112/Nectin (polyclonal, Novus), and pan-cytokeratin (clone AE1/AE3, Dako). Tissue slides were scanned using the Vectra 3.0 automated quantitative pathology imaging system (Akoya Biosciences). Images were spectrally unmixed with inForm tissue analysis software (Akoya Biosciences), and component TIFFs were exported for whole slide analysis using QuPath software.

### Statistics.

Baseline patient and disease characteristics were summarized using descriptive statistics. Descriptive statistics (counts/percentages, median/range) and graphical techniques (e.g., swimmer, waterfall plots) were used to summarize and assess toxicity, disease response, and PD-L1 NK cell persistence outcome data. All calculations were performed using R v3.6.3. By design, this interim assessment did not include formal statistical hypothesis testing; therefore, formal statistical comparisons, tests of association/differences, and *P* values are not provided.

### Study approval.

The present studies in humans or with human material (City of Hope IRB no. 20684) were reviewed and approved by the City of Hope Institutional Review Board. This study is registered with ClinicalTrials.gov (NCT05334329) and was conducted as per the Declaration of Helsinki and Good Clinical Practice. An appointed external committee (Data Safety Monitoring Board) provided oversight on collected data and patients’ safety. All patients provided written informed consent to participate in this study.

### Data availability.

Values for all data points in Figure 3 are reported in the Supporting Data Values file. Some data generated in this study may not be publicly available due to HIPAA-protected information that could compromise patient privacy or consent but are available upon reasonable request to the corresponding authors.

## Author contributions

MAVC provided study design, clinical care, writing and editing of the manuscript. XL and CA provided data acquisition, recording and safety support, and statistical support. JMP provided study design and statistical support. HM, ERB, and IB performed clinical and laboratory monitoring. CC and OB worked on the investigational new drug application and study monitoring. LT, TWS, CE, and LZ provided correlative science technical assistance. JV contributed to writing and editing the manuscript. SJF provided clinical care and study advisement. MAC provided conceptualization, correlative science, writing and editing of the manuscript. JY provided conceptualization, correlative science, and editing of the manuscript.

## Supplementary Material

Supplemental data

ICMJE disclosure forms

Supporting data values

## Figures and Tables

**Table 1:**
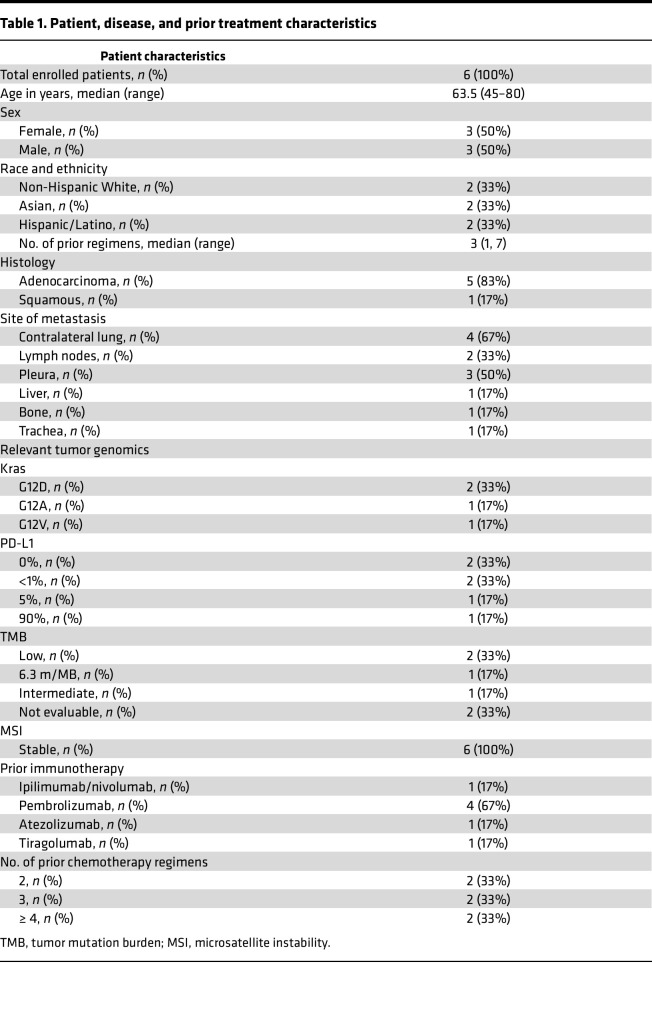
Patient, disease, and prior treatment characteristics

**Table 2:**
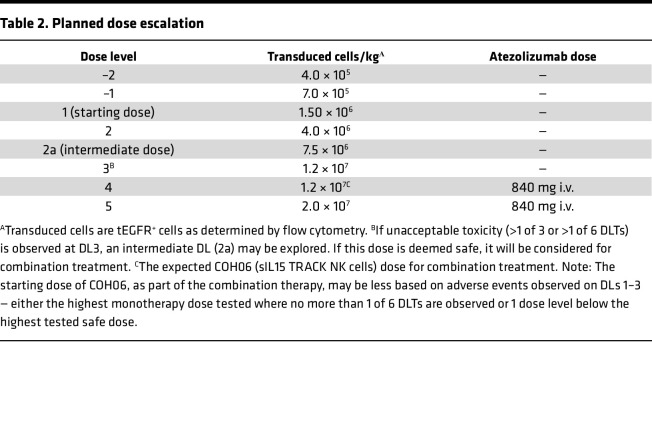
Planned dose escalation

**Table 3:**
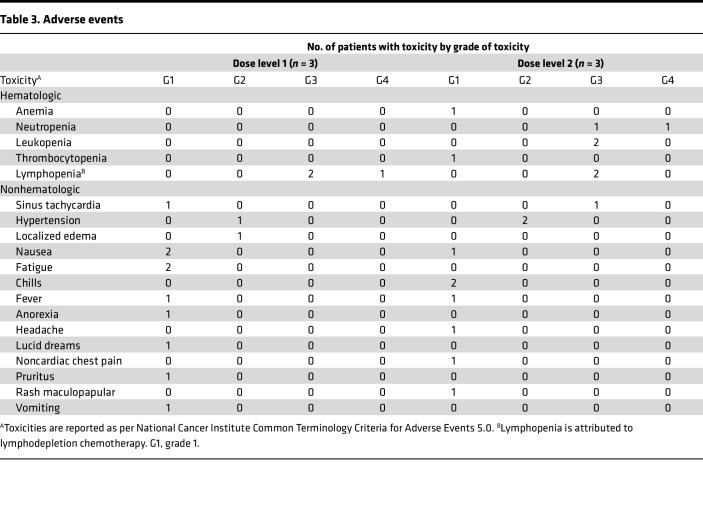
Adverse events

## Version 1 02/04/2025

In-Press Preview

## Version 2 03/24/2025

Electronic publication

## References

[1] Bray F (2024). Global cancer statistics 2022: GLOBOCAN estimates of incidence and mortality worldwide for 36 cancers in 185 countries. CA Cancer J Clin.

[2] Malhotra J (2016). Risk factors for lung cancer worldwide. Eur Respir J.

[3] Molina JR (2008). Non-small cell lung cancer: epidemiology, risk factors, treatment, and survivorship. Mayo Clin Proc.

[4] Garon EB (2015). Pembrolizumab for the treatment of non-small-cell lung cancer. N Engl J Med.

[5] Borghaei H (2015). Nivolumab versus docetaxel in advanced nonsquamous non-small-cell lung cancer. N Engl J Med.

[6] Brahmer J (2015). Nivolumab versus docetaxel in advanced squamous-cell non-small-cell lung cancer. N Engl J Med.

[7] Hellmann MD (2017). Nivolumab plus ipilimumab as first-line treatment for advanced non-small-cell lung cancer (CheckMate 012): results of an open-label, phase 1, multicohort study. Lancet Oncol.

[8] Jaiyesimi IA (2024). Therapy for stage IV non-small cell lung cancer with driver alterations: ASCO Living Guideline, Version 2023.3. J Clin Oncol.

[9] Dong W (2019). The Mechanism of Anti-PD-L1 antibody efficacy against PD-L1-negative tumors identifies NK cells expressing PD-L1 as a cytolytic effector. Cancer Discov.

[10] Bai R (2022). Burgeoning exploration of the role of natural killer cells in Anti-PD-1/PD-L1 therapy. Front Immunol.

[11] Villegas FR (2002). Prognostic significance of tumor infiltrating natural killer cells subset CD57 in patients with squamous cell lung cancer. Lung Cancer.

[12] Takanami I (2001). The prognostic value of natural killer cell infiltration in resected pulmonary adenocarcinoma. J Thorac Cardiovasc Surg.

[13] Cornel AM (2020). MHC Class I downregulation in cancer: underlying mechanisms and potential targets for cancer immunotherapy. Cancers (Basel).

[14] Freeman AJ (2019). Natural killer cells suppress T cell-associated tumor immune evasion. Cell Rep.

[15] Zhang X (2014). B7-H6 expression in non-small cell lung cancers. Int J Clin Exp Pathol.

[16] Liu H (2019). Role of NKG2D and its ligands in cancer immunotherapy. Am J Cancer Res.

[17] Oyama R (2022). CD155 expression and its clinical significance in non-small cell lung cancer. Oncol Lett.

[18] Molfetta R (2020). CD155: A multi-functional molecule in tumor progression. Int J Mol Sci.

[19] Ghadially H (2017). MHC class I chain-related protein A and B (MICA and MICB) are predominantly expressed intracellularly in tumour and normal tissue. Br J Cancer.

[20] Chan CJ (2009). DNAM-1/CD155 interactions promote cytokine and NK cell-mediated suppression of poorly immunogenic melanoma metastases. J Immunol.

[21] Brandt CS (2009). The B7 family member B7-H6 is a tumor cell ligand for the activating natural killer cell receptor NKp30 in humans. J Exp Med.

[22] Lin M (2020). Pembrolizumab plus allogeneic NK cells in advanced non-small cell lung cancer patients. J Clin Invest.

[23] Liu E (2020). Use of CAR-transduced natural killer cells in CD19-positive lymphoid tumors. N Engl J Med.

[24] Lu T (2024). Preclinical evaluation of off-the-shelf PD-L1^+^ human natural killer cells secreting IL15 to treat non-small cell lung cancer. Cancer Immunol Res.

[25] Villalona-Calero MA (2023). A phase 1 trial of umbilical cord blood–derived tumor-reactive PD-L1^+^ natural killer cells engineered to express soluble IL-15 (TRACK-NK) in patients with non–small-cell lung cancer (NSCLC) refractory to PD-1/PD-L1 inhibitors. J Clin Oncol.

[26] Teng KY (2022). Off-the-shelf prostate stem cell antigen-directed chimeric antigen receptor natural killer cell therapy to treat pancreatic cancer. Gastroenterology.

[27] Carson WE (1994). Interleukin (IL) 15 is a novel cytokine that activates human natural killer cells via components of the IL-2 receptor. J Exp Med.

[28] Carson WE (1997). A potential role for interleukin-15 in the regulation of human natural killer cell survival. J Clin Invest.

[29] Cooper MA (2002). In vivo evidence for a dependence on interleukin 15 for survival of natural killer cells. Blood.

[30] Dubois S (2002). IL-15Ralpha recycles and presents IL-15 In trans to neighboring cells. Immunity.

[31] Kobayashi H (2005). Role of trans-cellular IL-15 presentation in the activation of NK cell-mediated killing, which leads to enhanced tumor immunosurveillance. Blood.

[32] Fehniger TA (2001). Fatal leukemia in interleukin 15 transgenic mice follows early expansions in natural killer and memory phenotype CD8^+^ T cells. J Exp Med.

[33] Kennedy MK (2000). Reversible defects in natural killer and memory CD8 T cell lineages in interleukin 15-deficient mice. J Exp Med.

[34] Guimond M (2010). In vivo role of Flt3 ligand and dendritic cells in NK cell homeostasis. J Immunol.

[35] Yu P (2012). Simultaneous inhibition of two regulatory T-cell subsets enhanced Interleukin-15 efficacy in a prostate tumor model. Proc Natl Acad Sci U S A.

[36] Guzman G (2023). CAR-T therapies in solid tumors: opportunities and challenges. Curr Oncol Rep.

[37] Lee SM (2023). First-line atezolizumab monotherapy versus single-agent chemotherapy in patients with non-small-cell lung cancer ineligible for treatment with a platinum-containing regimen (IPSOS): a phase 3, global, multicentre, open-label, randomised controlled study. Lancet.

[38] Chen Y (2013). Effects of MICA expression on the prognosis of advanced non-small cell lung cancer and the efficacy of CIK therapy. PLoS One.

[39] Sivori S (2019). Human NK cells: surface receptors, inhibitory checkpoints, and translational applications. Cell Mol Immunol.

[40] Mansour AG (2023). Off-the-shelf CAR-engineered natural killer cells targeting FLT3 enhance killing of acute myeloid leukemia. Blood Adv.

[41] Lee DW (2019). ASTCT consensus grading for cytokine release syndrome and neurologic toxicity associated with immune effector cells. Biol Blood Marrow Transplant.

[42] https://www.federalregister.gov/documents/2006/11/28/E6-20129/guidance-for-industry-gene-therapy-clinical-trials-observing-subjects-for-delayed-adverse-events.

