# Supplementary material for: Interim report on engineered NK cell trial in lung cancer refractory to immune checkpoint inhibitors
Source: JCI Insight. 2025 Feb 4;10(6):e186890. doi: 10.1172/jci.insight.186890 (PMC11949060; doi:10.1172/jci.insight.186890)
Supplement: Supplemental data [file jciinsight-10-186890-s312.pdf]

Supplementary Information

First-in-Human Trial of Engineered NK Cells in Lung Cancer Refractory to Immune Checkpoint Inhibitors

Miguel A Villalona-Calero[1,2]*, Lei Tian[3], Xiaochen Li[4], Joycelynne M. Palmer[3,4,8], Claudia Aceves[5], Hans Meisen[5], Catherine Cortez[5], Timothy W. Synold[5], Colt Egelston[5], Jeffrey VanDeusen[6], Ivone Bruno[7], Lei Zhang[7], Eliezer Romeu-Bonilla[7], Omer Butt[7], Stephen J. Forman[8], Michael A. Caligiuri[8]*, Jianhua Yu[2,8,9,10]*

Affiliations:

From (1) The Department of Medical Oncology and Experimental Therapeutics, Beckman Research Institute and Comprehensive Cancer Center, City of Hope, Los Angeles, CA USA. (2) Current address: Department of Medicine, Division of Hematology Oncology, Chao Family Comprehensive Cancer Center, University of California at Irvine, CA USA. (3) Hematologic Malignancies Research Institute, Department of Hematology and Hematopoietic Stem Cell Transplantation, City of Hope National Medical Center, Los Angeles, CA USA. (4) Division of Biostatistics, Department of Computational and Quantitative Medicine, City of Hope Los Angeles, CA USA. (5) Beckman Research Institute, City of Hope, Los Angeles CA USA. (6) Avita Health Systems, Galion, OH USA. (7) CytoImmune Therapeutics, Los Angeles, CA USA. (8) Beckman Research Institute and Comprehensive Cancer Center, City of Hope, Los Angeles, CA USA. (9) Institute for Precision Cancer Therapeutics and Immuno-Oncology, Chao Family Comprehensive Cancer Center, (10) The Clemons Family Center for Transformative Cancer Research, University of California, Irvine, California CA USA

*Corresponding authors

Miguel A. Villalona-Calero
Hematology-Oncology
UCI Chao Family CCC
Suite 400, Room 413
200 S. Manchester Ave.
Orange, CA 92868
+1 (714) 456-8100
mavilla4@uci.edu

Michael A. Caligiuri
Hematology
City of Hope
Kaplan 3
1500 E Duarte Rd
Los Angeles CA 91010
+1 (626) 218-4328
mcaligiuri@coh.org

Jianhua Yu
Hematology-Oncology
UCI Chao Family CCC
Suite 400, Room 413
200 S. Manchester Ave.
Orange, CA 92868
+1 (949) 824-3926
jianhuay@hs.uci.edu

Conflict of interest statement: Drs. Yu and Caligiuri are co-founders and shareholders of CytoImmune Therapeutics; both serve as consultants to CytoImmune Therapeutics, a financial sponsor of this study. Drs. Yu and Caligiuri have a patent filed through City of Hope for sIL15_TRACK NK cells (US20220249564A1). All other authors have no conflict of interest.

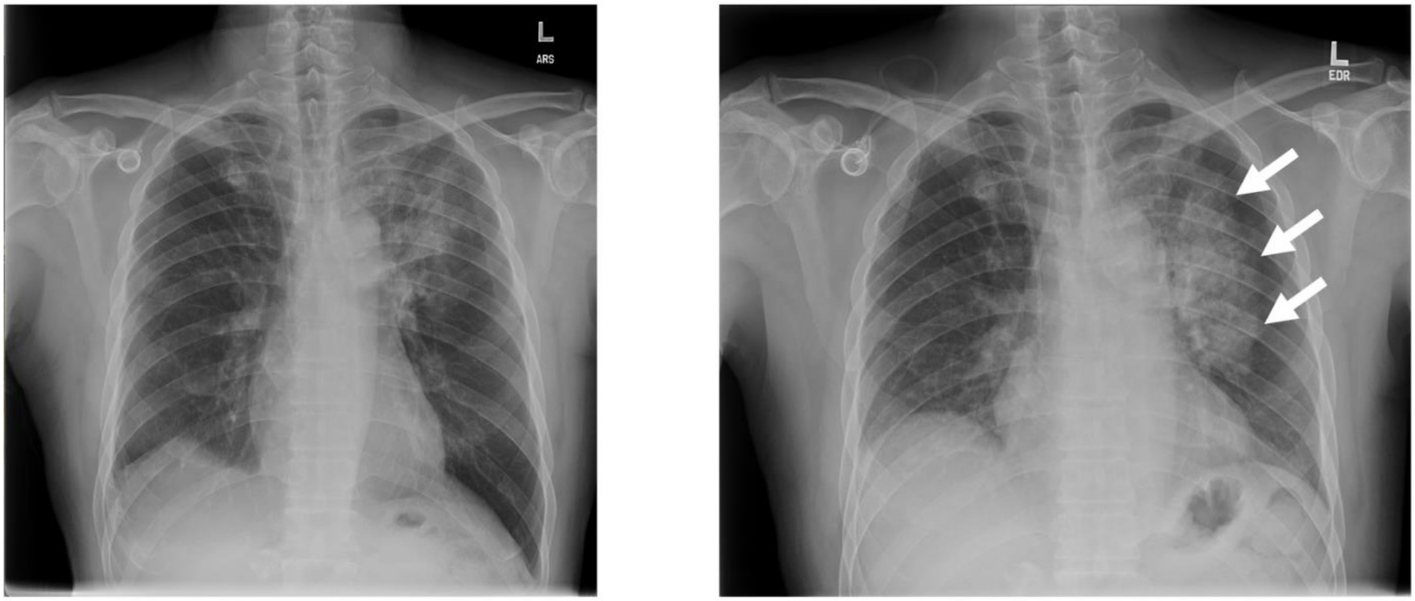

Supplementary Figure 1. The chest X-Ray on the left shows a baseline film of patient 005 before starting cycle 2 of sIL15_TRACK NK cell infusions at a dose of 4.0 x 10^6 transduced cells/kg. The patient became acutely short of breath following the third weekly infusion of cycle 2 and a chest X-Ray was again obtained demonstrating a new infiltrate (white arrows) that subsequently improved spontaneously within seven days without the onset of fever or additional intervention.

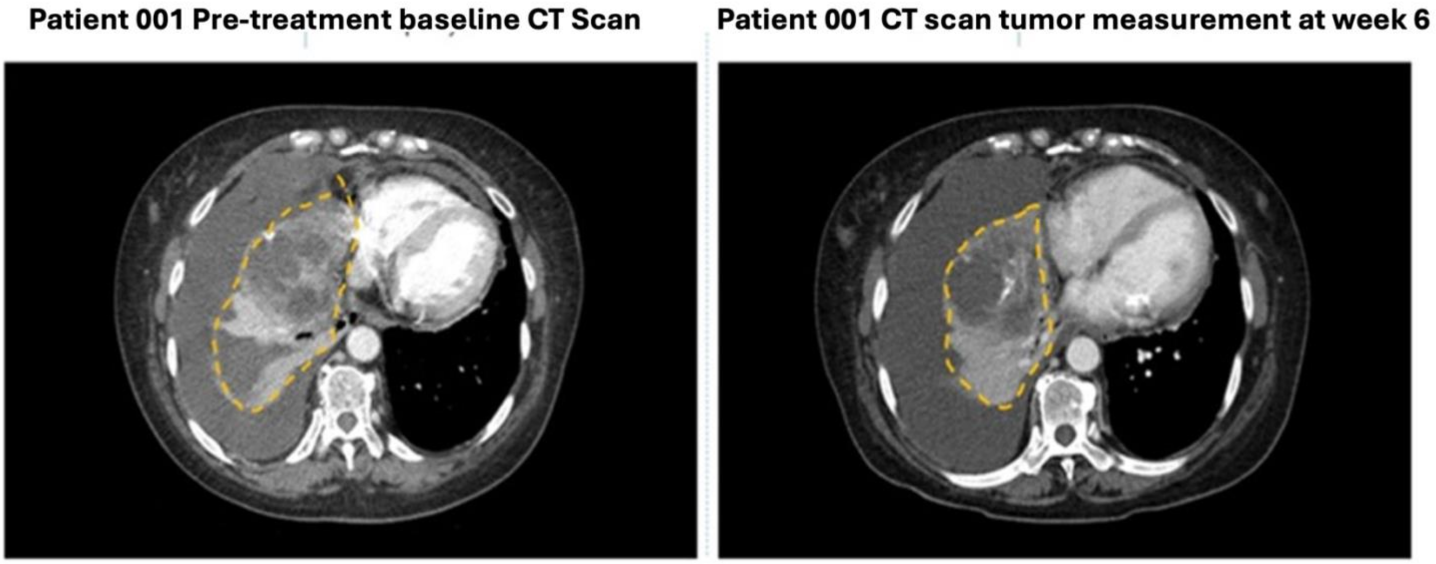

Supplementary Figure 2. The CT scan on the left shows a baseline tumor measurement of patient 001 immediately prior to starting cycle 1 of sIL15_TRACK NK cell infusions at a dose of 1.5 x 10^6 transduced cells/kg. The yellow dashed line indicates the size of the target lesion. The CT scan on the right shows an approximately 12% reduction in tumor volume when measured 6 weeks after the start of cycle 1 and 2 weeks after completion of cycle 1. Patient 001 died unexpectedly of a cardiac event with a diagnosis of COVID while on cycle 2 of sIL15_TRACK NK cells without evidence of progression.

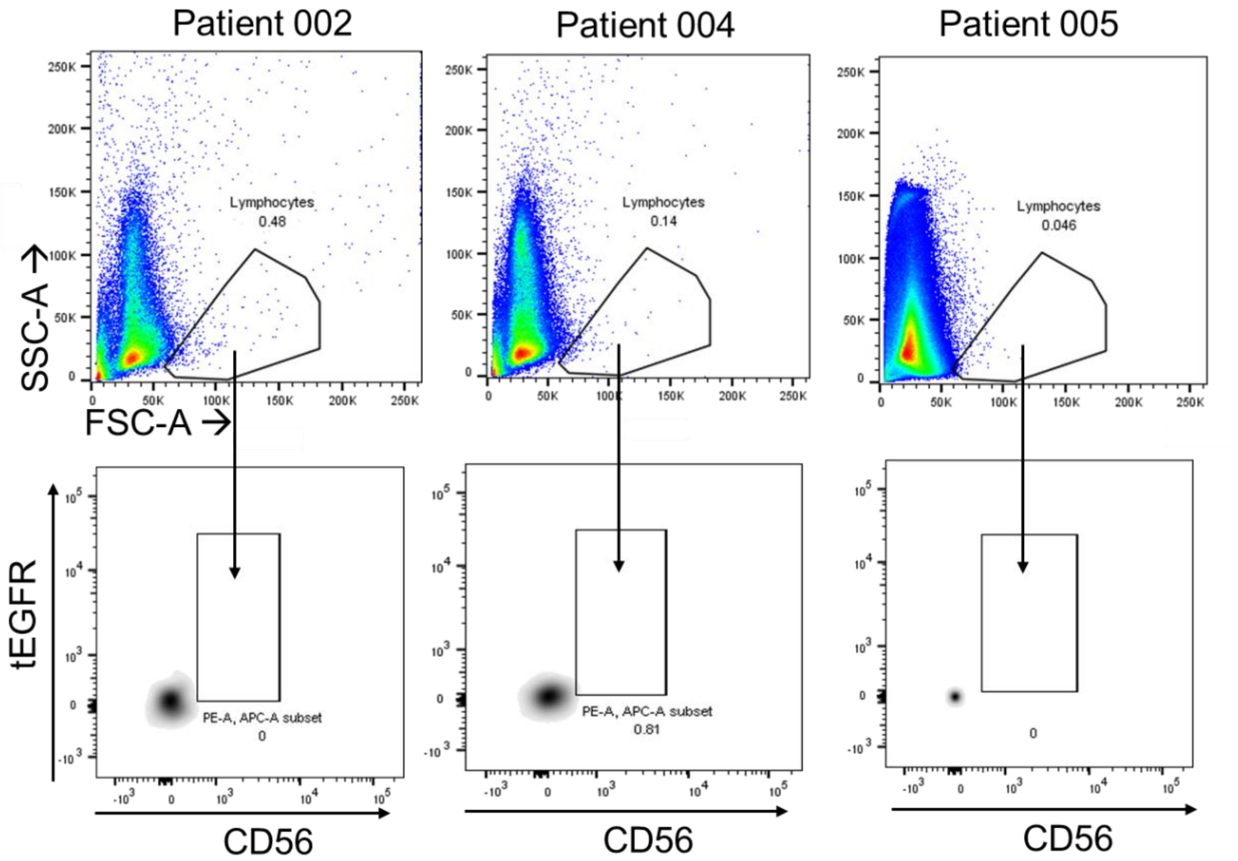

Supplementary Figure 3. Three patient lung biopsies were collected between 1 and 7 days following the 4th infusion of sIL15_TRACK NK cells and digested to single cell suspension using collagenase I and DNase I. Cells were stained with antibodies against CD56 and EGFR and assessed by flow cytometry to identify CD56+tEGFR+ sIL15_TRACK NK cells within the lymphocyte gate. Very few (< 0.5%) lymphocytes were collected in biopsies from each of the three patients shown here, yielding no CD56+tEGFR+ sIL15_TRACK NK cells.

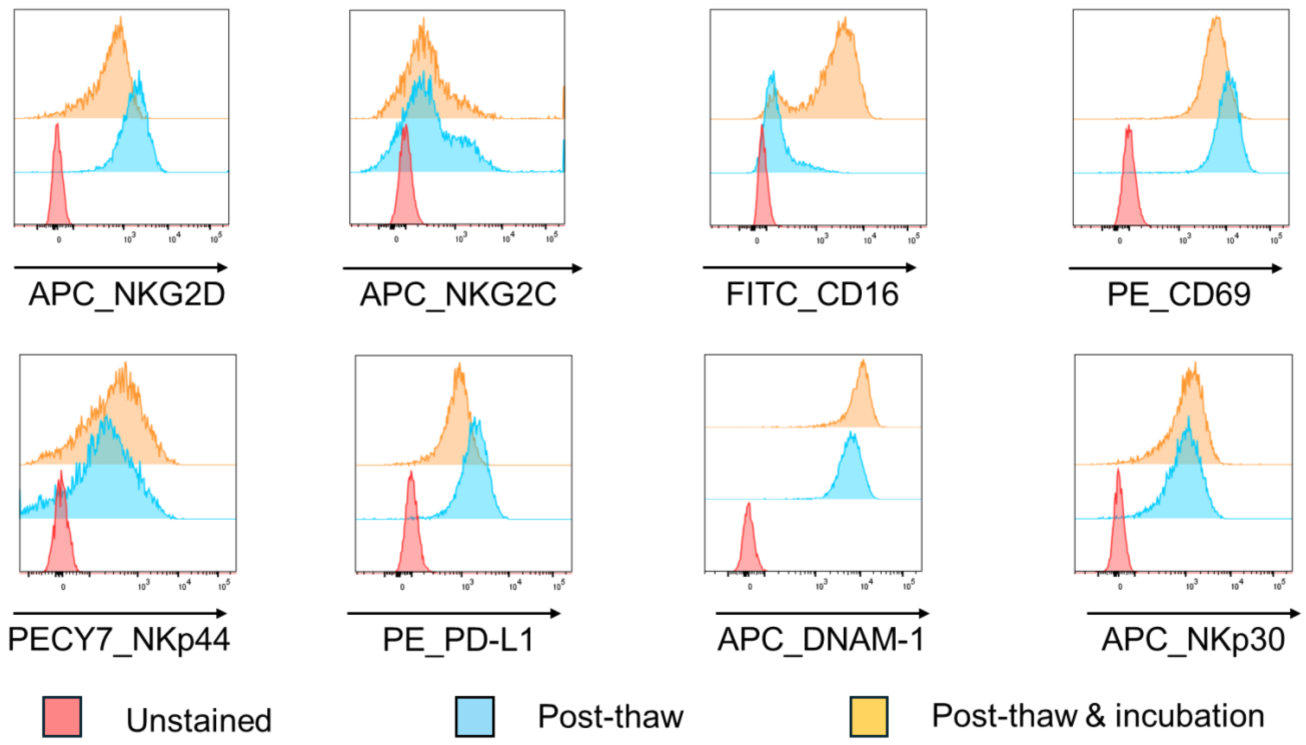

Supplementary Figure 4. Phenotype of cryopreserved sIL15_TRACK NK cells immediately post-thaw and two days after thaw and after 48 hours of in vitro incubation at 37°C with low dose IL-2.
